# Excellent removal of knob-into-hole bispecific antibody byproducts and impurities in a single-capture chromatography

**DOI:** 10.1186/s40643-022-00562-y

**Published:** 2022-07-04

**Authors:** Serene W. Chen, Kong Meng Hoi, Farouq Bin Mahfut, Yuansheng Yang, Wei Zhang

**Affiliations:** 1 Downstream Processing Group, Bioprocessing Technology Institute, Agency for Science, Technology and Research, Singapore, Singapore; 2 Cell Line Development Group, Bioprocessing Technology Institute, Agency for Science, Technology and Research, Singapore, Singapore

**Keywords:** Bispecific antibody, Knob-into-hole, Chromatography purification, Low-pH wash, Product-related impurities, Hole–hole homodimer, Process-related impurities, Aggregation propensity

## Abstract

Bispecific antibodies (bsAbs) are therapeutically promising due to their ability to bind to two different antigens. However, the bsAb byproducts and impurities, including mispaired homodimers, half-antibodies, light chain mispairings, antibody fragments and high levels of high molecular weight (HMW) species, all pose unique challenges to their downstream processing. Here, using two knob-into-hole (KiH) constructs of bsAbs as model molecules, we demonstrate the excellent removal of bsAb byproducts and impurities in a single Protein A chromatography under optimized conditions, including hole–hole homodimer mispaired products which are physicochemically very similar to the target bsAbs and still present even with the use of the KiH format, though at reduced levels. The removal occurs through the incorporation of an intermediate low-pH wash step and optimal elution conditions, achieving ~ 60% monomeric purity increase in a single Protein A step, without the introduction of sequence-specific bsAb modifications to specifically induce differential Protein A binding. Our results also suggest that the higher aggregation propensity of bsAbs may cause aggregation during the column process, hence an optimization of the appropriate loading amount, which may be lower than that of monoclonal antibodies (mAbs), is required. With the use of loading at 50% of 10% breakthrough (QB10) at 6-min residence time, we show that an overall high monomer purity of 92.1–93.2% can be achieved with good recovery of 78.4–90.6% within one capture step, which is a significant improvement from a monomer purity of ~ 30% in the cell culture supernatant (CCS). The results presented here would be an insightful guidance to all researchers working on the purification process development to produce bispecific antibodies, especially for knob-into-hole bispecific antibodies.

**Graphical Abstract:**

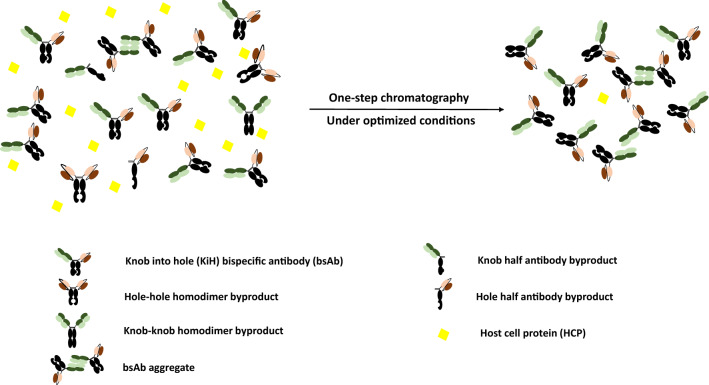

## Introduction

Bispecific antibodies (bsAbs) possess great therapeutic potential in addressing the multifactorial nature of complex diseases through the recognition and binding to two different antigens. In contrast to their parental monoclonal antibodies (mAbs) that bind to a single target, bsAbs can be used for a wide range of applications, such as the simultaneous blockage of two different mediators, selective retargeting of effector mechanisms to defined disease-associated sites, or as drug delivery vectors (Konterman 2005; Baeuerle and Reinhardt 2009; Chames and Baty 2009; Kontermann 2012; Brinkmann and Kontermann 2017; Labrijn et al. 2019). A myriad of different bsAb formats have so far been reported, with three approved bsAbs—blinatumomab (Blincyto) (Gökbuget et al. 2018; Kantarjian et al. 2017), emicizumab (Hemlibra) (Oldenburg et al. 2017) and amivantamab (Rybrevant) (Neijssen et al. 2021; Syed 2021)—currently in the market and many more in clinical development (Kontermann 2005, 2012; Baeuerle and Reinhardt 2009; Chames and Baty 2009; Brinkmann and Kontermann 2017; Labrijn et al. 2019).

The therapeutic advantage brought about by this class of antibodies through its increased valency is nevertheless often accompanied by a higher level of impurities, therefore posing unique challenges to their downstream processing. An overall higher aggregation propensity has been reported for various formats of bsAbs including fragment-based bsAbs that lack the Fc region as well as symmetric bsAbs (Garber 2014; Taki et al. 2015; Andrade et al. 2019; Michaelson et al. 2009; Schanzer et al. 2011), with up to 50% of aggregates and even expression as inclusion bodies observed in some cases (Jakobsen 2011; Vallera and Miller 2017). BsAb-specific byproducts, including fragments such as ½ antibodies (bsAbs lacking a HC and LC) as well as heavy chain (HC) and light chain (LC) mispaired products, represent another major source of impurities that can cause downstream processing burdens, with undesired mispaired products accounting for up to 90% of the total mass if left to pair randomly (Klein et al. 2012).

One of the strategies employed to reduce such mispaired products is the generation of bsAbs via the knob-into-hole (KiH) approach (Ridgway et al. 1996), which is designed to favor the formation of the target heterodimer bsAb over the mispaired products. However, hole–hole homodimerization can still occur at low levels, with the occurrence of the knob–knob homodimers being rarer due to the inherent steric hindrance of the knobs. In contrast to this approach which does not necessarily provide a clear purification strategy to remove mispaired products that do occur, a different approach is to generate bsAbs with differential binding affinity to specific affinity resins on each arm. Examples of which include the introduction of mutations to alter Protein A-binding affinities, such as the chimeric Fc sequence (Fc*) (Tustian et al. 2016), or by incorporating parts of antibodies which do not bind to Protein A with those that have a high affinity, such as the rat/mouse quadroma-derived bsAb (Lindhofer et al. 1995), as well as to design bsAbs with different types or number of light chains on each arm, such as the Kappa (К) and/or Lambda (λ) light chains (Qin et al. 2020; Fischer et al. 2015). While this approach generally provides a direct strategy for the removal of the mispaired byproducts during subsequent downstream processing steps, cellular energy spent on making the unwanted mispaired products lowers the overall productivity.

One of the most commonly employed affinity chromatography steps for bsAbs, as is the case for mAbs, is that of Protein A chromatography (Chen and Zhang 2021; Li et al. 2020). In particular, differential Protein A affinity chromatography has been proposed to be able to separate the target bsAb from the undesired heavy chain mispaired products through the use of a pH gradient or multi-step pH elutions (Tustian et al. 2016; Lindhofer et al. 1995; Smith et al. 2015; Zwolak et al. 2017; Zwolak et al. 2017a, b; Skegro et al. 2017; Ollier et al. 2019), particularly for bsAbs generated with modifications made to alter the Protein A-binding affinity between target bsAb and that of mispaired products. The use of salts has also been reported to improve the separation resolution between the target bsAb and binding mispaired homodimers (Tustian et al. 2016) as well as ½ antibodies (Chen et al. 2020). With regard to resin selection, engineered Protein A affinity ligands that lack VH binding, such as MabSelect SuRe (Tustian et al. 2016), have been proposed to be useful in preventing a reduced avidity difference between the target bsAb and undesired bound homodimers due to VH3–Protein A interactions (Sasso et al. 1991, 1989). Compared to the MabSelect SuRe resin, the latest generation of Protein A affinity chromatography resin from Cytiva—MabSelect PrismA exhibits an even higher dynamic binding capacity and alkaline stability, and the ligand has conversely been engineered to possess an enhanced binding to the VH3 domain (Cytiva 2021), which has recently been utilized to separate the desired bsAb target from a byproduct which has lost one Fab arm (Zhang et al. 2021).

Here, using two KiH bsAb constructs without sequence-specific modifications to their Protein A-binding affinity and MabSelect PrismA resin, we report the removal of both high molecular weight (HMW) and low molecular weight (LMW) impurities, including mispaired products and half-antibodies, through the incorporation of an intermediate-pH wash step and optimal elution conditions in Protein A affinity chromatography. Within one capture step, we demonstrate a reduction of high molecular weight (HMW) species from above 30% to ~ 5% and low molecular weight (LMW) species from above 30% to ~ 1%, yielding >  ~ 90% and >  ~ 78% monomer purity and recovery, respectively.

## Materials and methods

Unless otherwise stated, all buffers, salts and reagents were purchased from Merck Millipore. MabSelect PrismA (Cytiva) resin as well as Tricorn™ series columns (Cytiva) was kindly provided by Cytiva.

### bsAb culture

Stably transfected CHO K1 cells lines producing FabscFv-KiH and Fab_2_scFv-KiH were generated by site-specific integration of the plasmid vectors carrying genes encoding light chain (LC), heavy chain (HC) and scFv-Fc (for FabscFv-KiH) or VH-CH1-scFv-Fc (for Fab_2_scFv-KiH). CH3 domains in the HC and scFv-Fc/VH-CH1-scFv-Fc were engineered to form a knob (through mutations of S354C:T366W) and a hole (through mutations of Y349C:T366S:Y407V), respectively, to facilitate the heterodimeric Fc pairing based on a previous study (Merchant 1998). In FabscFv-KiH, the VH and VL in scFv was connected through a flexible linker (G4S)3 which was further linked to the Fc through a G4 linker. The engineered Fc consisted of IgG1 positions 221 to 447 based on the Eu numbering system. In Fab2scFv-KiH, VH-CH1 was linked to the N-terminus of scFv-Fc through a G4S linker.

The stably transfected cell lines were grown in a protein-free medium consisting of HyQ PF (Cytiva) and CD CHO (Thermo Fisher Scientific) at 1:1 ratio and supplemented with 1 g/L sodium carbonate (Sigma), 6 mM glutamine (Sigma), and 0.1% Pluronic F-68 (Thermo Fisher Scientific) in 50 mL tubespin (TPP) in a humidified Kuhner shaker (Adolf Kühner AG) with 8% CO_2_ at 37 °C. To produce FabscFv-KiH or Fab_2_scFv-KiH, 300 mL of cell culture at viable cell density of 3 × 105 cells/mL were inoculated into the 600 mL tubespin (TPP) in the humidified Kuhner shaker (Adolf Kühner AG) with 8% CO_2_ at 37 °C. 30 mL of Ex-Cell Advanced CHO Feed 1 (with glucose) (SAFC, Sigma) were added at day 3, 5, 7 9 and 11. Cell density and viability of each culture were monitored at day 3, 5, 7, 9, 11 and 14 using the Vi-Cell XR viability analyzer (Beckman Coulter). d-Glucose concentration in the culture medium was quantified using Nova bioprofile 100plus analyzer (Nova Biomedical). When the glucose concentration in the media drops below 2 g/L, d-glucose (Sigma) was added to the culture to adjust the glucose concentration above 6 g/L. Culture supernatant was harvested at day 14 and centrifuged to remove cells before proceeding to purification.

### AKTA™ chromatography

1 mL and 5 mL of MabSelect PrismA (Cytiva) resin were packed in Tricorn™ series columns (Cytiva) with a bed height of 5.1 cm and 6.4 cm, respectively, with experiments conducted on an AKTA™ Avant 25 (Cytiva). All columns were equilibrated with 100 mM sodium phosphate, 150 mM NaCl, pH 7.2, before loading the appropriate amount of CCS. A 3 CV wash of 50 mM Na-citrate, pH 6.0 was performed after loading of CCS for all experiments. All elution buffers contain 50 mM Na-citrate at their respective pH between 6.0 and 3.0. The pH values of collected eluates were measured using an external pH probe (Mettler Toledo), where necessary.

### Antibody concentration and purity analysis

The antibody concentration and purity were analyzed by HPLC-SEC using a TSK_gel_ G3000SW_XL_ column (7.8 mm i.d. × 30 cm; Tosoh Bioscience) at a flow rate of 0.6 mL/min. The mobile phase consisted of 0.2 M l-arginine, 0.05 M MES, 5 mM EDTA, 0.05% sodium azide (w/w), pH 6.5. The UV absorbance was monitored at 280 nm, with the resultant concentrations determined based on the area under the peaks as compared to a calibration curve obtained using standard samples. The amount of aggregates and fragments present in the sample was calculated using the area of peaks which eluted before and after the monomeric peak, respectively.

Due to the high amount of impurities and low titre of bsAb target molecule in the CCS, the initial bsAb titre determination was obtained by subtracting the background signal, which was obtained by taking the difference between the monomeric peak integration and monomeric baseline integration in the flowthrough (FT). For dynamic binding capacity (DBC) analysis, the monomeric bsAb concentration was estimated by performing a monomeric peak integration of the respective FT fractions. The breakthrough percentage was obtained by considering the percentage of monomer concentration in the FT relative to that in the CCS load. The mass balance analysis was performed by comparing the area of the respective species obtained from HPLC-SEC multiplied by the respective volume obtained from the AKTA system.

As a complementary approach to HPLC-SEC analysis, non-reducing SDS-PAGE gels (4–15% Criterion™ TGX Stain-Free™ Protein Gel, Bio-rad) were also used to evaluate the purity of the samples, according to manufacturer’s instructions. A total protein amount of 0.3 µg was loaded per lane based on Bradford assay (Thermo Fisher Scientific), with staining performed with eLuminol™ (GeneCopoeia).

## Results

### Preliminary evaluation of the performance of Protein A chromatography for bsAb molecules

**Fig. 1:**
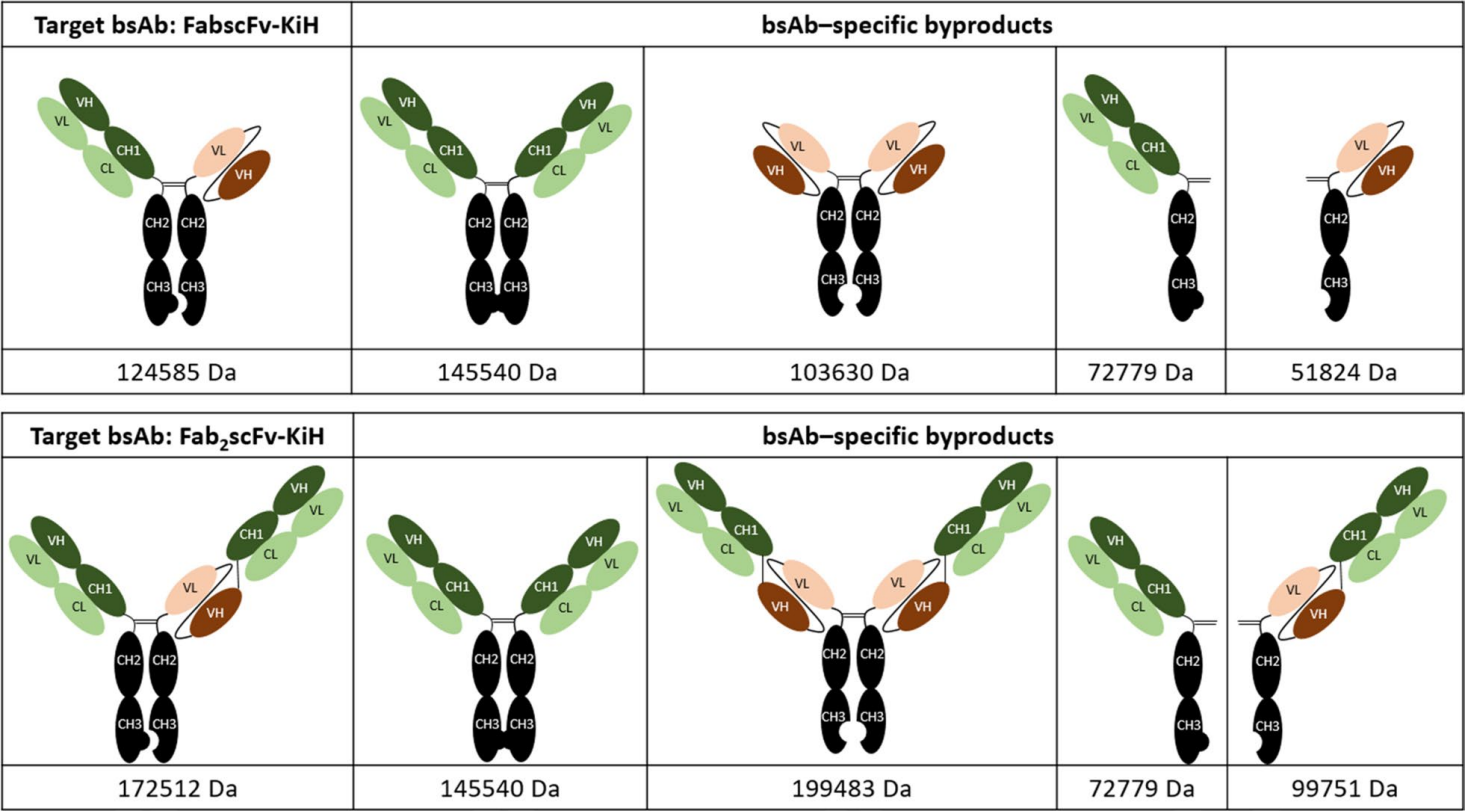
Schematic representation of the model asymmetric bsAbs along with their possible major byproducts. FabscFv-KiH and Fab_2_scFv-KiH are the model asymmetric bsAbs used in this study, and their possible major byproducts include the mispaired homodimers and half-antibodies. The corresponding molecular weight indicated below each species was calculated based on its respective protein sequence

**Fig. 2:**
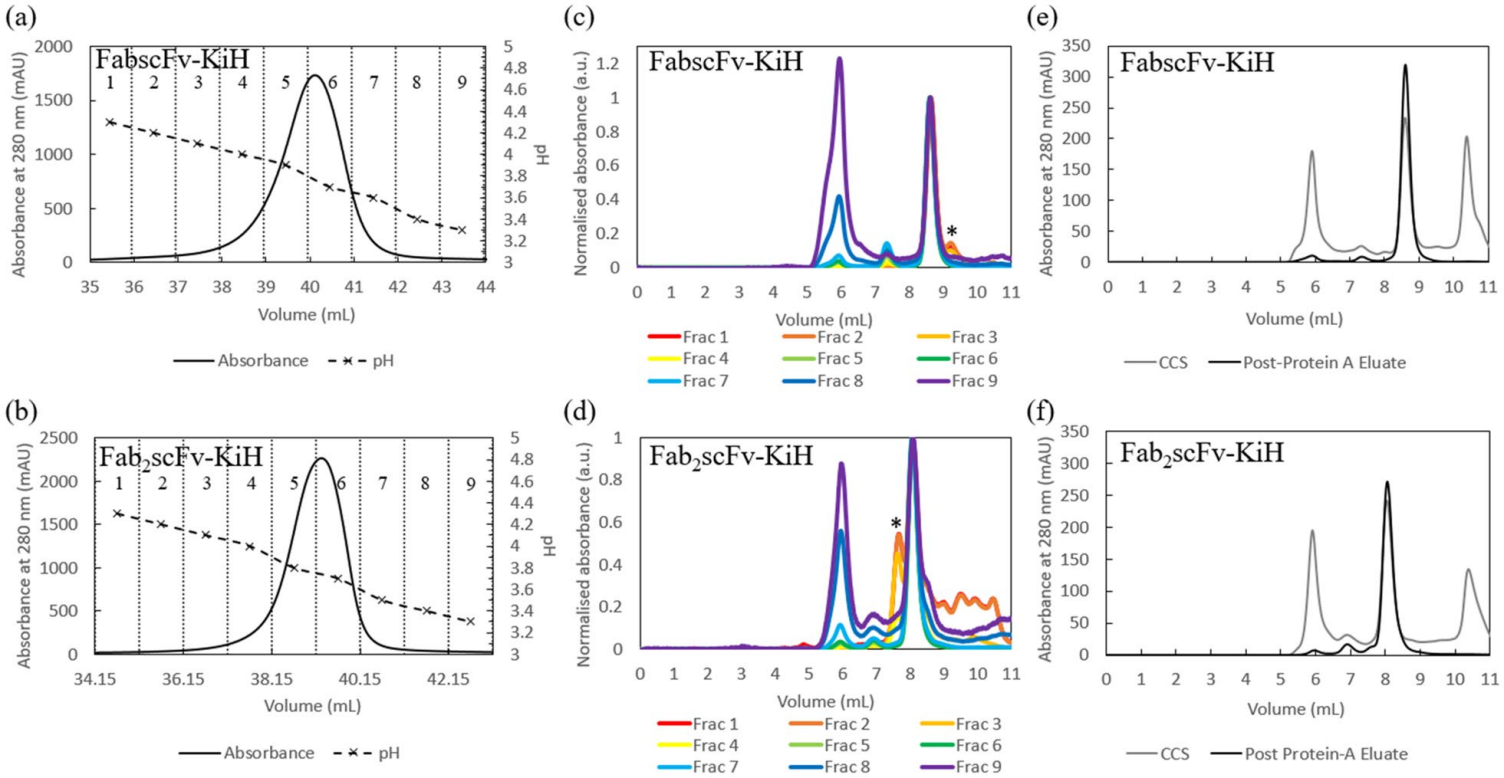
AKTA chromatograms and HPLC-SEC chromatograms of elute fractions of MabSelect PrismA runs. AKTA chromatogram of FabscFv-KiH (**a**) and Fab_2_scFv-KiH (**b**), with a pH gradient elution from pH 6.0 to pH 3.0 in 25 CV using MabSelect PrismA resin. The pH values indicated here represent the pH of each fraction measured by an external pH probe, with each fraction number indicated at the top of the chromatogram. HPLC-SEC chromatogram of FabscFv-KiH (**c**) and Fab_2_scFv-KiH (**d**) of each fraction of the eluates with absorbance values normalized to that of the monomeric peak, where each absorbance value was divided by the highest absorbance value of the monomeric peak. The likely hole–hole homodimeric mispaired products are indicated with*. The purity profile of the pooled post-Protein A eluate of all 9 fractions with respect to the CCS is also illustrated for FabscFv-KiH (**e**) and Fab_2_scFv-KiH (**f**)

We first set out to evaluate the performance of MabSelect PrismA Protein A affinity chromatography as a capture step for the bsAb KiH construct, using 2 model bsAb molecules—FabscFv-KiH and Fab_2_scFv-KiH as illustrated in Fig. 1, along with the possible bsAb-specific mispaired homodimeric products and half-antibodies. In order to probe the optimal pH for step elution, a low loading of 9 – 10 mg/mL resin (R) was used for this preliminary evaluation with a pH gradient elution from pH 6.0 (50 mM Na-citrate) to 3.0 (50 mM Na-citrate) in 25 column volumes (CVs) at 2-min residence time (Fig. 2). Na-citrate buffer was chosen as elution buffer in our study as it is the recommended conditions for MabSelect PrismA Affinity chromatography in its user manual (Cytiva 2020a, b). In this way, the high molecular weight (HMW) species were significantly reduced from ~ 35% to ~ 7% and the low molecular weight (LMW) species were significantly reduced from ~ 35% to below 3% in both FabscFv-KiH and Fab_2_scFv-KiH Protein A eluates when the whole peak was collected (Table 1, Fig. 2). It is worth noting that while a significant proportion of HMW species eluted out at later fractions of lower pH for both FabscFv-KiH and Fab_2_scFv-KiH, the earlier fractions that eluted at higher pH consist of a significant proportion of LMW impurities (Fig. 2 c, d). Interestingly, in both cases, species with molecular weight close to that of the target bsAb as highlighted in the HPLC-SEC chromatograms in Fig. 2 c and d with a*, likely to correspond to the hole–hole homodimeric mispaired product, eluted at higher pH values.

**Table 1:** Preliminary run for MabSelect PrismA, where 9–10 mg of bsAb monomer containing CCS was loaded per mL of resin, with 3 CVs of 50 mM Na-citrate pH 6.0 wash step followed by their respective elution conditions

		Elution conditions	Monomer concentration (mg/mL)	Monomer recovery (%)	Purity (%)		
					HMW	Mono	LMW
FabscFv-KiH	CCS	–	0.70	–	34.9	28.9	36.2
	MabSelect PrismA eluate	pH gradient elution from pH 6.0 to pH 3.0 in 25 CVs	1.02	101.1	8.5	90.9	0.6
		2-step elution:					
		pH 3.8 mock pool	0.21	44.4	1.7	97.6	0.8
		pH 3.6 mock pool	0.50	50.1	6.0	94.0	0.1
		2-step elution:					
		pH 3.6 mock pool	0.78	94.6	6.7	92.3	0.9
		pH 3.4 mock pool	0.02	0.8	61.8	26.7	11.5
Fab_2_scFv-KiH	CCS	–	0.78	–	34.3	30.4	35.3
	MabSelect PrismA eluate	pH gradient elution from pH 6.0 to pH 3.0 in 25 CVs	0.98	92.9	10.9	87.2	1.8
		2-step elution:					
		pH 3.8 mock pool pH 3.6 mock pool	0.24	51.2	5.1	89.8	5.1
			0.52	43.9	6.8	91.1	2.1
		2-step elution:					
		pH 3.6 mock pool	1.11	93.5	7.7	89.4	2.9
		pH 3.4 mock pool	0.02	0.6	47.4	24.4	28.2

**Fig. 3:**
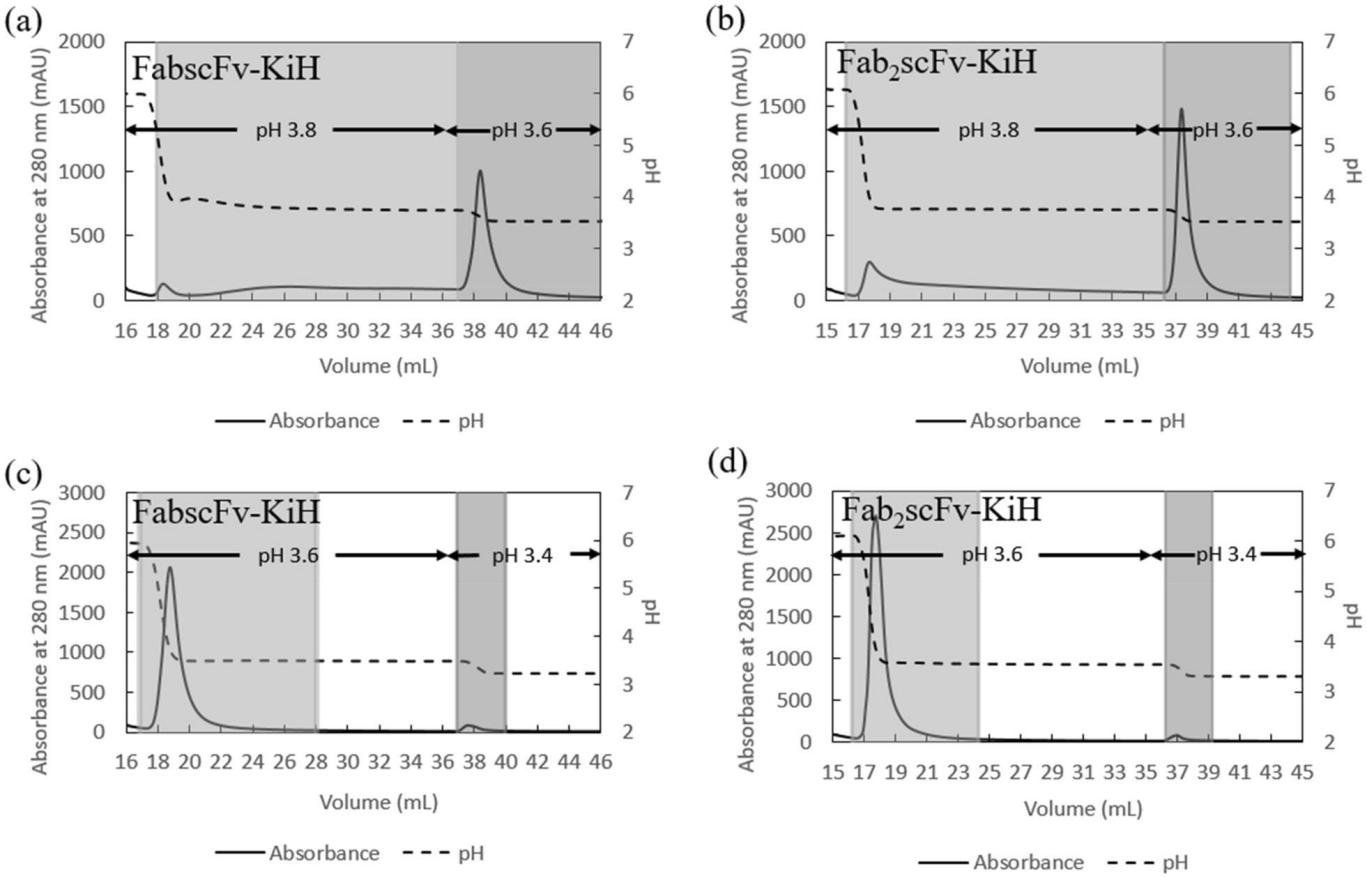
AKTA chromatograms of MabSelect PrismA runs with a 2-step pH elution. A 2-step elution of pH 3.8 and pH 3.6 for FabscFv-KiH (**a**) and Fab_2_scFv-KiH (**b**), respectively, and a 2-step elution of pH 3.6 and pH 3.4 for FabscFv-KiH (**c**) and Fab_2_scFv-KiH (**d**), respectively, with the higher and lower pH eluate analyzed highlighted in light and dark grey, respectively

As the main peak of both molecules eluted between pH 3.7–3.9 as measured by an external pH probe, a 2-step elution at pH 3.8 and 3.6 was subsequently performed for 20 CV and 10 CV, respectively (Fig. 3 a, b). It was observed for both molecules that both pH eluates obtained at pH 3.8 and pH 3.6 yielded a very high monomer purity of >  ~ 90%; nevertheless, the recovery at pH 3.8 was low whereas the remaining bsAb was eluted at pH 3.6 (Table 1), suggesting that a pH of at least 3.6 or lower was required to effectively elute the target bsAbs. In order to investigate both yield and purity at pH 3.6 and if the elution pH should be lowered further, a 2-step elution at pH 3.6 and 3.4 was therefore performed for 20 CV and 10 CV, respectively (Fig. 3 c, d). The step elution first performed at pH 3.6 yielded both high monomer purity (> ~ 90%) and high monomer recovery (> ~ 90%) (Table 1), with < 1% of the target bsAbs obtained with low purity in the pH 3.4 eluate, thus confirming pH 3.6 as the optimal elution pH.

### Investigation of optimal loading conditions of bsAbs on MabSelect PrismA resin

**Fig. 4:**
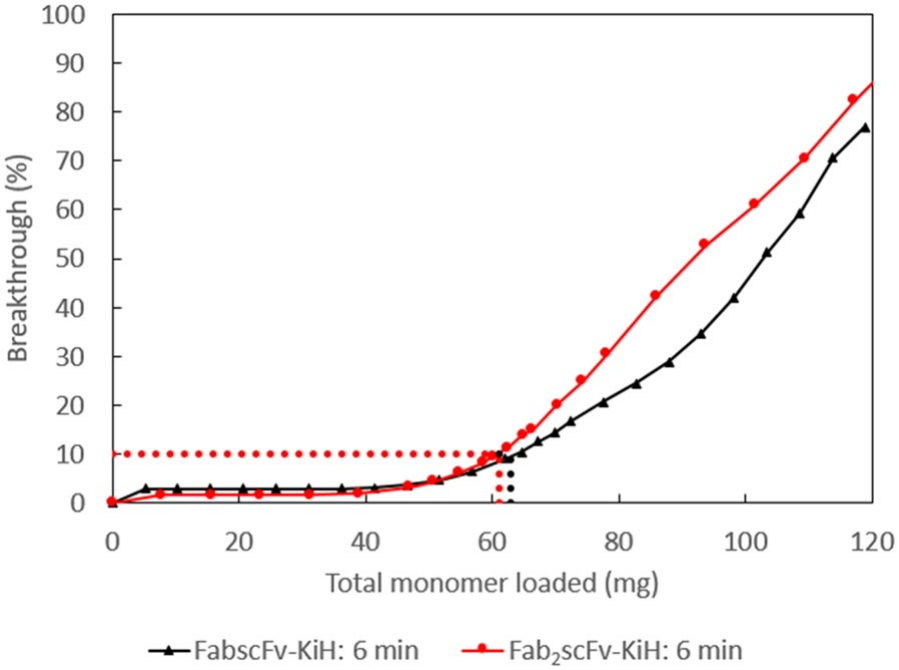
Breakthrough curve study at 6-min residence time for FabscFv-KiH (black) and Fab_2_scFv-KiH (red)

Given the promising nature of MabSelect PrismA resin in obtaining high bsAb target purity and recovery, the optimal loading conditions were next investigated in order to develop an industrially applicable process. The DBC studies were performed at the industrially relevant 6-min residence time, so as to determine the optimal loading amounts for these two bsAbs on the MabSelect PrismA resin. By loading CCS onto the resin and monitoring the amount of monomer obtained in the FT, the breakthrough curves were obtained and the DBC was determined at 10% breakthrough (QB10) to be 63 mg/mL and 61 mg/mL for FabscFv-KiH and Fab_2_scFv-KiH, respectively (Fig. 4).

**Fig. 5:**
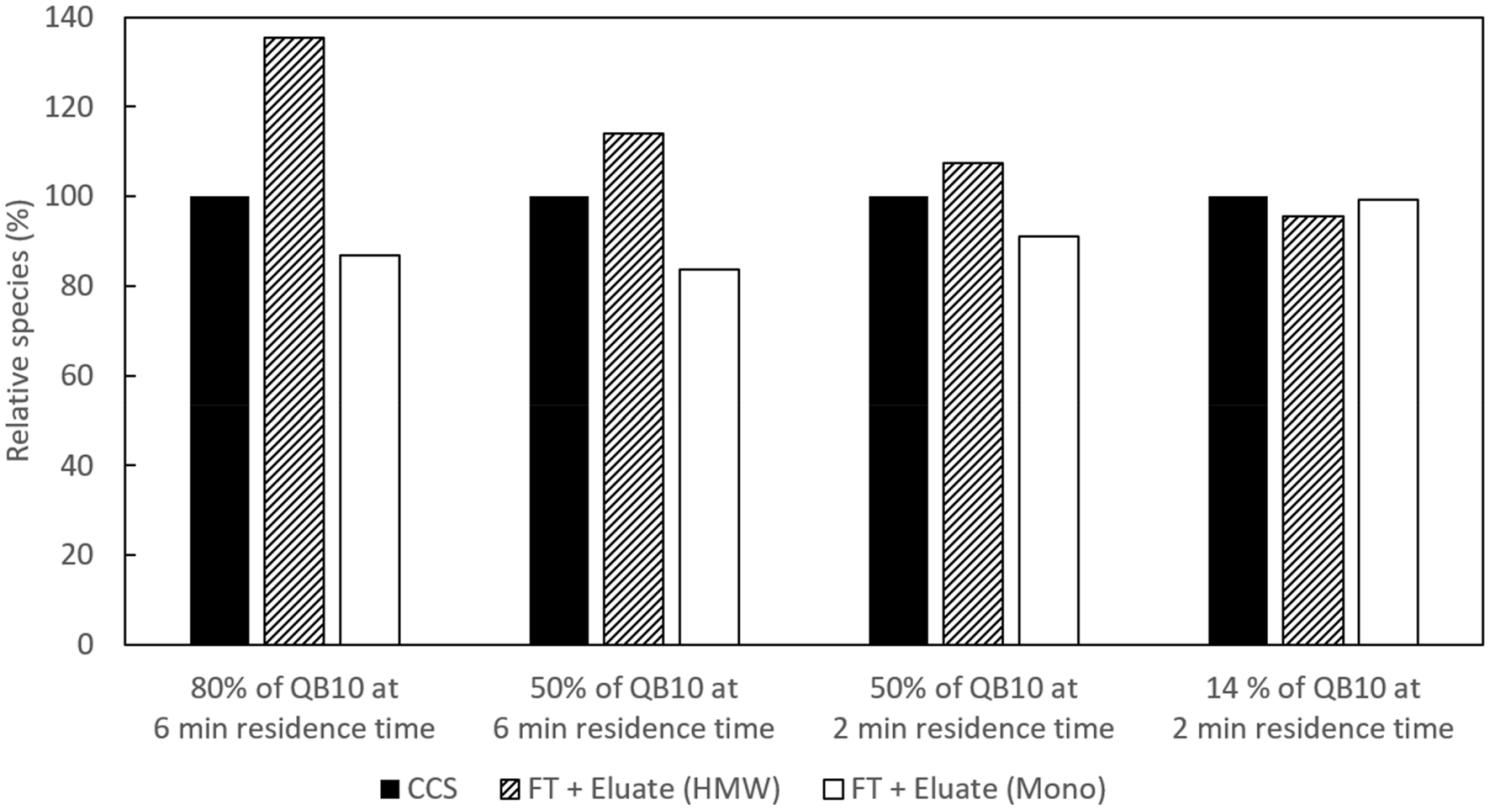
Mass balance analysis of relative HMW and monomer species. Mass balance analysis of relative HMW and monomer species in CCS versus the total in FT and post-Protein A eluate at the different load and residence time studies. The black bar represents HMW amount in CCS as 100% when calculating the relative amount of HMW in FT + eluate, and it represents Mono amount in CCS as 100% when calculating the relative amount of Mono in FT + eluate

Using the previously ascertained optimal pH elution condition, a run was first performed with loading at 80% of QB10 at 6-min residence time for FabscFv-KiH. The monomer recovery and monomer purity obtained decreased to 82.4% and 85.3%, respectively, with the percentage HMW species doubling to 14.2% (Table 2), compared to the low loading of 9 mg/mL-R (14% of QB10) 2-min residence time (Table 1). A mass balance analysis of the amount of HMW and monomeric species in the CCS versus FT and eluate indicates that while the relative amount of HMW and monomer remains fairly constant when loading at 14% of QB10 at 2-min residence time, there was a large increase in HMW species at higher load, i.e. 80% of QB10 at 6-min residence time along with a concomitant decrease in monomeric species in the FT and eluate sample compared to the CCS, thus suggesting the presence of aggregation during the column process (Fig. 5).

**Table 2:** Effect of different loading amounts and residence time on the purity profile and recovery of post-Protein A eluates obtained with pH 3.6 elution

		Loading conditions			Monomer concentration (mg/mL)	Monomer recovery (%)	Purity (%)		
		QB10 (%)	Residence time (min)	Total loading time (h)			HMW	Mono	LMW
FabscFv-KiH	CCS	–	–	–	0.52	–	29.5	28.3	42.2
	MabSelect PrismA eluate	80	6	9.7	2.77	82.4	14.2	85.3	0.5
		50	6	6.0	2.59	90.4	11.0	88.5	0.5
		50	2	6.0	2.59	90.4	11.2	88.3	0.5
Fab_2_scFv-KiH	CCS	–	–	–	0.72	–	31.1	33.5	35.4
	MabSelect PrismA eluate	50	6	4.2	2.14	91.2	8.4	89.1	2.5

In order to determine if this can be attributed to the increased loading amount or residence time, two additional runs were performed both with a load of 50% of QB10 but one at 6-min residence time and the other at 2-min residence time. The monomer recovery and monomer purity obtained in these 2 runs were similar at 90.4% and 88.3–88.5%, respectively (Table 2). This suggests that an increase in loading amount, rather than an increase in residence time, leads to an increase in HMW species. This is further corroborated by the mass balance analysis, where a similar slight increase in HMW species and slight decrease in monomeric species was observed at a load corresponding to 50% of QB10 at 6-min and 2-min residence times. A load of 50% of QB10 at 6-min residence time was therefore selected as the optimal loading amount. This load was also verified for the Fab_2_scFv-KiH molecule, where it was found that a high monomer recovery and purity of 91.2% and 89.1% were maintained, respectively (Table 2), both of which were comparable to that obtained at low loading (Table 1).

### Improvement in purity with an additional intermediate-pH wash step with final load conditions

**Fig. 6:**
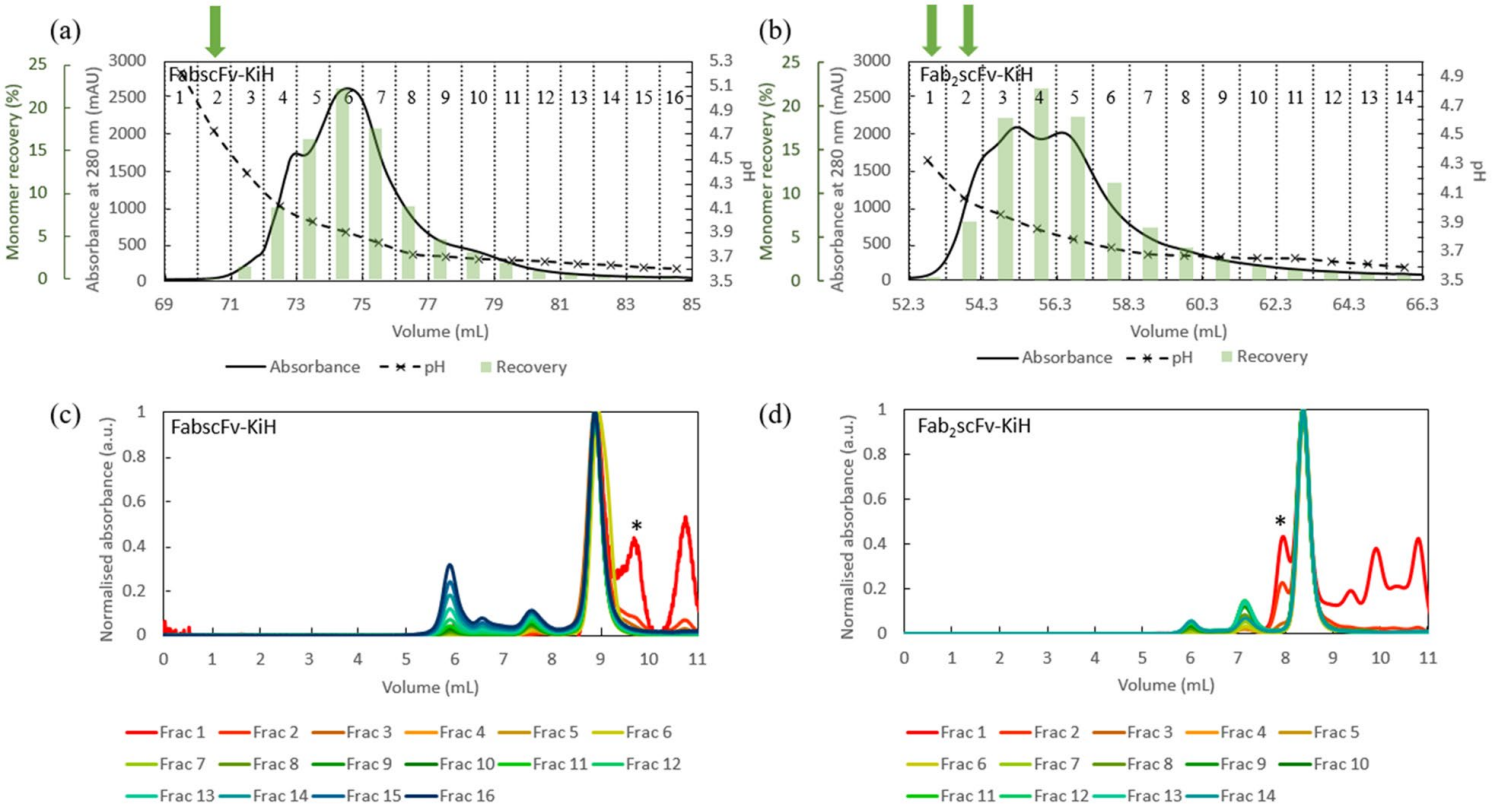
AKTA chromatograms and HPLC-SEC chromatograms of elute fractions of MabSelect PrismA runs. AKTA chromatogram of the 5 CV gradient elution from pH 6.0 to pH 3.6 with a 15 CV hold at pH 3.6, with the point corresponding to pH 4.7 for FabscFv-KiH (**a**) as well as pH 4.3 and 4.1 for Fab_2_scFv-KiH (**b**) indicated with green arrows. The pH values indicated here represent the pH of each fraction measured by an external pH probe, with each fraction number indicated at the top of the chromatogram. HPLC-SEC chromatogram of FabscFv-KiH (**c**) and Fab_2_scFv-KiH (**d**) of each fraction of the eluates with absorbance values normalized to that of the monomeric peak, where each absorbance value was divided by the highest absorbance value of the monomeric peak. The likely hole–hole homodimeric mispaired products are indicated with*

As the high amount of product-related impurities may pose a challenge for the subsequent polishing resins, it was of importance to investigate the capability of the Protein A resin to remove as much of these impurities as possible. Using the industrially relevant load of 50% of QB10 at 6-min residence time, a 5 column volume (CV) gradient elution from pH 6.0 to pH 3.6 was therefore performed, with a 15 CV hold at pH 3.6 at the end, so as to determine a suitable pH condition for an intermediate wash step. As in the case of the low loading, LMW species as well as the possible hole–hole mispaired species eluted at higher pH and HMW species eluted at lower pH compared to the main peak (Fig. 6). A majority of the hole–hole mispaired product and LMW species relative to the target molecule appeared to elute at pH 4.7 for FabscFv-KiH and pH 4.3 and 4.1 for Fab_2_scFv-KiH as measured by the external pH probe, with pH 4.1 eluting ~ 7% of the target Fab_2_scFv-KiH monomer. The impact of introducing an intermediate-pH wash condition at the respective pH values for 10 CV for FabscFv-KiH and Fab_2_scFv-KiH was therefore investigated.

**Fig. 7:**
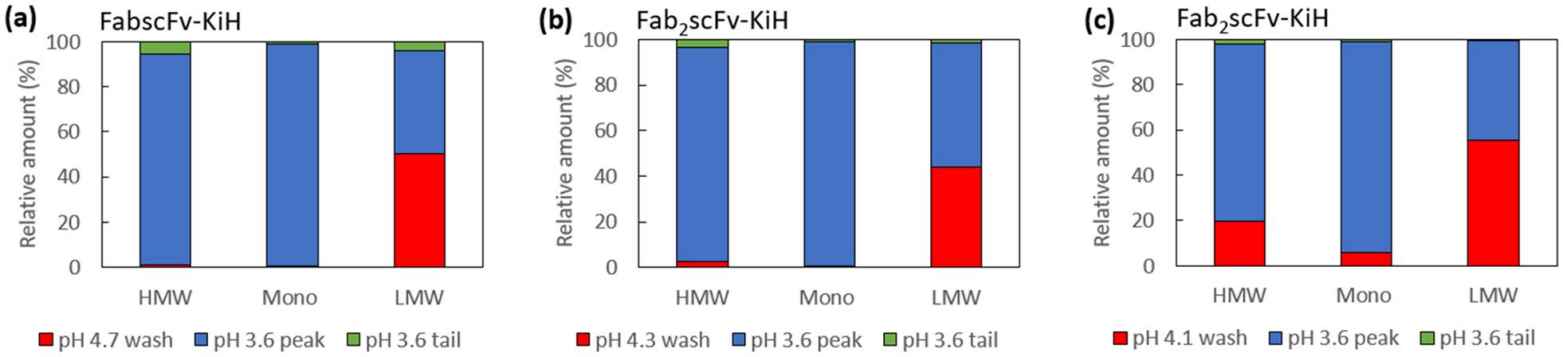
Analysis of the relative amount of HMW, monomer and LMW species in the intermediate low-pH wash, pH 3.6 eluate peak and tail process fractions for the Protein A run of **a** FabscFv-KiH with pH 4.7 intermediate wash, **b** Fab_2_scFv-KiH with pH 4.3 intermediate wash and **c** Fab_2_scFv-KiH with pH 4.1 intermediate wash. The area under the HPLC-SEC curve for HMW, monomeric and LMW species was multiplied by the volume collected during the Protein A chromatography run for each process fractions. The resultant value obtained for each species was summed up across all process fractions and taken as 100%

**Fig. 8:**
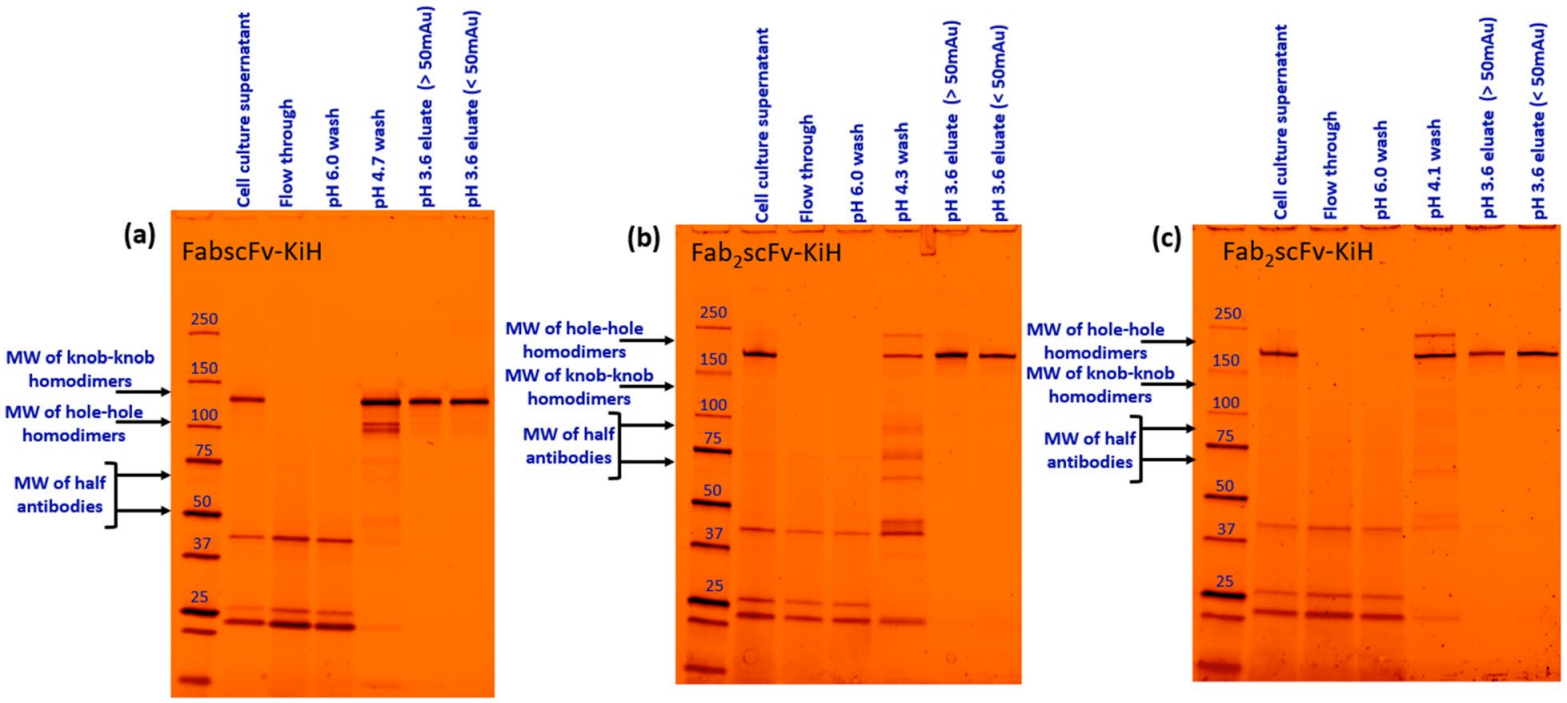
SDS-PAGE gel illustrating the purity profile for different steps of the Protein A runs. FabscFv-KiH (**a**) and Fab_2_scFv-KiH with pH 4.3 wash step (**b**) and pH 4.1 wash step (**c**)

In this way, a high monomer purity of 92.9% and 92.3% can be obtained for FabscFv-KiH and Fab_2_scFv-KiH using an intermediate-pH wash condition of pH 4.7 and 4.3, respectively, yielding recoveries of 90.6% and 86.6%, respectively (Table 3). As the LMW species for Fab_2_scFv-KiH is still rather high at 1.9% with a pH 4.3 intermediate wash, the effect of performing an intermediate-pH wash at pH 4.1 for 10 CV was further evaluated. It was observed that the LMW species can be further reduced by 0.5% under this condition, at the expense of ~ 8% recovery (Table 3). An analysis of the relative amount of HMW, monomer and LMW species obtained in the low-pH intermediate wash compared to that of the pH 3.6 peaks reflects the ability of the low-pH intermediate wash at removing LMW species for both molecules, with the lower pH 4.1 wash for Fab_2_scFv-KiH contributing towards the removal of HMW impurities as well (Fig. 7). Furthermore, it was observed that the omission of the pH 3.6 tail (UV_280_ < 50 mAU) enables the further removal of HMW species in all cases (Fig. 7). These results are corroborated by the SDS-PAGE gel analysis (Fig. 8), which shows that while a pH 6.0 wash removes non-specific host cell protein (HCP) binding as in the FT, the intermediate low-pH wash is indeed able to remove LMW species, including half-antibodies, as well as hole–hole homodimer products based on their expected molecular weight (Fig. 8). SDS-PAGE gel analysis together with HPLC-SEC further confirmed that for FabscFV-KiH, its HMW species most likely consist of heteroaggregates and knob–knob homodimers, while its LMW species most likely consist of hole–hole homodimers, half-antibodies and various HCPs. For Fab_2_scFV-KiH, its HMW species most likely consist of heteroaggregates and hole–hole homodimers, while its LMW species most likely consist of knob–knob homodimers, half-antibodies and various HCPs. Finally, the scalability of the process was evaluated by performing the run in a 5-mL column. The recovery and purity are maintained (Table 4), with < 3.5% and < 1.0% difference in recovery and purity, respectively, compared to that of a 1-mL column, hence reflecting the scalability of the process.

**Table 3:** The purity profile, monomer concentration and recovery of each step of the Protein A run for both FabscFv-KiH and Fab_2_scFv-KiH in 1-mL columns with a load of 50% of QB10 at 6-min residence time, with the introduction of an intermediate low-pH wash step

			Conditions	Monomer concentration (mg/mL)	Monomer recovery (%)	Purity (%)		
						HMW	Mono	LMW
1-mL column	FabscFv-KiH	CCS	–	0.69	–	30.8	35.5	33.8
		MabSelect PrismA	FT	0.02	2.4	–	–	–
			pH 6.0 wash	0.01	0.1	–	–	–
			*pH 4.7 wash*	0.03	1.2	7.6	48.2	44.2
			pH 3.6 peak (UV_280_ > 50 mAu)	3.65	90.6	6.7	92.9	0.4
			pH 3.6 tail (UV_280_ < 50 mAu)	0.04	1.2	29.7	67.7	2.6
	Fab_2_scFv-KiH	CCS	-	0.72	-	33.5	31.1	35.4
		MabSelect PrismA	FT	0.02	2.7	-	-	-
			pH 6.0 wash	0.01	0.1	-	-	-
			*pH 4.3 wash*	0.02	0.7	6.1	18.3	75.6
			pH 3.6 peak (UV_280_ > 50 mAu)	2.04	86.6	5.8	92.3	1.9
			pH 3.6 tail (UV_280_ < 50 mAu)	0.06	1.1	16.8	78.9	4.3
		MabSelect PrismA	FT	0.01	1.9	-	-	-
			pH 6.0 wash	0.01	0.1	-	-	-
			*pH 4.1 wash*	0.15	4.9	17.8	62.3	19.9
			pH 3.6 peak (UV_280_ > 50 mAu)	1.56	78.7	6.4	92.2	1.4
			pH 3.6 tail (UV_280_ < 50 mAu)	0.07	1.1	12.2	87.1	0.7

**Table 4:** Validation runs for both FabscFv-KiH and Fab_2_scFv-KiH in 5-mL columns

			Conditions	Monomer concentration (mg/mL)	Monomer recovery (%)	Purity (%)		
						HMW	Mono	LMW
5-mL column	FabscFv-KiH	CCS	–	0.69	–	30.8	35.5	33.8
		MabSelect PrismA	FT	0.02	2.8			
			pH 6.0 wash	0.01	0.1			
			pH 4.7 wash	0.02	0.6	5.4	51.5	43.1
			pH 3.6 peak (UV_280_ > 50 mAu)	5.00	91.1	5.7	93.9	0.4
			pH 3.6 tail (UV_280_ < 50 mAu)	0.03	1.3	26.0	71.7	2.3
	Fab_2_scFv-KiH	CCS	–	0.72	–	33.5	31.1	35.4
		MabSelect PrismA	FT	0.01	2.0	–	–	–
			pH 6.0 wash	0.01	0.1	–	–	–
			*pH 4.3 wash*	0.01	0.4	7.6	3.4	89.0
			pH 3.6 peak (UV_280_ > 50 mAu)	2.74	89.8	6.1	92.1	1.8
			pH 3.6 tail (UV_280_ < 50 mAu)	0.06	2.0	14.2	84.3	1.5
		MabSelect PrismA	FT	0.01	2.0	–	–	–
			pH 6.0 wash	0.01	0.1	–	–	–
			*pH 4.1 wash*	0.34	11.9	14.9	72.9	12.2
			pH 3.6 peak (UV_280_ > 50 mAu)	2.92	78.4	6.5	92.1	1.4
			pH 3.6 tail (UV_280_ < 50 mAu)	0.03	1.2	51.3	46.0	2.7

## Discussion

Protein A is arguably the most commonly used affinity-based capture purification method in downstream processing of mAbs and has been employed for bsAbs as well (Chen and Zhang 2021; Li et al. 2020), due to the interaction between the Protein A and the Fc region of the target antibody as well as the VH region of the heavy chain for targets belonging to the VH3 gene family (Sasso et al. 1991, 1989). MabSelect PrismA has been reported in the product brochure to possess a DBC, as determined at 10% breakthrough using a ~ 5 mg/mL human IgG sample, of ~ 80 mg human IgG/mL resin at 6-min residence time (Cytiva 2021; Cytiva 2020a, b; Cytiva 2022). Here, our results of 61–63 mg/mL at 6-min residence time for FabscFv-KiH and Fab_2_scFv-KiH, though slightly lower, are remarkable, considering the low titre and purity of the CCS. This shows that MabSelect PrismA is indeed a suitable and powerful resin for the purification of bispecific antibodies. As bsAbs in various formats have been reported to possess higher aggregation propensities (Garber 2014; Taki et al. 2015; Andrade et al. 2019; Michaelson et al. 2009; Schanzer et al. 2011), this can have important implications for the optimal load amount in columns. While a load of 80% of QB10 may be commonly used for mAbs, here we observed that such a loading amount resulted in an increase in HMW species and reduced recovery of post-Protein A eluate, suggesting the presence of on-column aggregation or aggregation during elution likely due to the higher aggregation propensity of bsAbs. To circumvent this problem, a lower load at ~ 50% of QB10 was found to be able to yield good recoveries and purities. This highlights the importance of probing the optimal load amount for bsAbs in order to ensure both high yield and purity of post-Protein A eluate. Also, in line with previous observations that the HMW aggregate species elute at lower pH as compared to that of the target bsAb (Andrade et al. 2019), we observed that further HMW species elute later in the tail end of the pH 3.6 eluate, hence HMW species can be further reduced by omitting the pH 3.6 eluate tail with UV 280 signal < 50 mAU.

One of the frequently reported approaches for the downstream removal of bsAb mispaired products involves the use of differential Protein A affinity chromatography, where specific sequence modifications on one arm of the bsAb can lead to differential Protein A-binding affinity of the mispaired products as compared to the target bsAb (Tustian et al. 2016; Lindhofer et al. 1995; Smith et al. 2015; Zwolak et al. 2017; Zwolak et al. 2017a, b; Skegro et al. 2017; Ollier et al. 2019). Here, without the use of specific constructs designed to possess differential Protein A-binding avidity between the target molecule and the mispaired products, we demonstrate that hole–hole homodimer mispaired products can be removed using an intermediate low-pH wash. This may be attributed to the fact that the hole–hole homodimer is less well folded due to a lack of CH3 domain dimerization (Chen et al. 2019), therefore reducing the binding affinity to the resin. This was observed to be true for both hole–hole mispaired products present in the two different KiH bsAb molecules.

Moreover, the VH region of both arms for both FabscFV-KiH and Fab_2_scFV-KiH molecules used in this study belongs to VH3, so both our bsAb molecules and their byproducts including hole–hole homodimers can bind to MabSelect PrismA via VH_3_–Protein A interaction. The conformational difference between bsAb molecules and their byproducts, especially at VH_3_ region, could increase the binding affinity difference which led to the effective byproducts removal by applying low-pH intermediate wash step. MabSelect SuRe could possibly provide good separation as well, but without additional contribution from VH3–Protein A interaction, the resolution might not be as good as MabSelect PrismA.

In addition to the homodimer mispaired products, fragments represent another set of impurities in bsAb cultures. Here, we demonstrate that the half-antibodies in both FabscFv-KiH and Fab_2_scFv-KiH which possess less Fc and VH3 regions compared to the target bsAb elute at pH values higher than that of the target molecule, along with other LMW species. This is in line with previous reports (Zhang et al. 2021; Cytiva) demonstrating the capability of Protein A chromatography at the removal of LMW species. Depending on the efficiency of the subsequent polishing steps, we also show that LMW species can be further reduced with the introduction of an even lower intermediate-pH wash of pH 4.1 instead of pH 4.3 for Fab_2_scFv-KiH, at the expense of monomer recovery.

Furthermore, for FabscFv-KiH format bsAb, as its hole–hole homodimer and hole half-antibody byproducts do not contain LC constant region, so it is highly possible that they can potentially be removed by subdomain-specific affinity resins such as KappaSelect. KappaSelect affinity chromatography could be a good alternative approach to remove byproducts of bsAb format which is LC constant region free on one of its arms.

## Conclusions

In conclusion, using MabSelect PrismA and two KiH bsAb molecules, we demonstrate the effective removal of both HMW and LMW species, including the mispaired homodimers and half-antibodies, in a single Protein A affinity chromatography step, achieving high purity and recovery of 92.1–93.2% and 78.4–90.6%, respectively, for this class of challenging biotherapeutics. These results illustrate the suitability of Protein A chromatography for bsAb purification, and importantly demonstrate its ability for the removal of bsAb-specific byproducts without the use of specific design constructs.

## Data Availability

All data generated or analyzed during this study are included in this published article.

## Abbreviations

bsAbs: Bispecific antibodies
HMW: High molecular weight
KiH: Knob-into-hole
mAbs: Monoclonal antibodies
QB10: 10% Breakthrough
CCS: Cell culture supernatant
HC: Heavy chain
LC: Light chain
Fc*: Chimeric Fc sequence
LMW: Low molecular weight
FT: Flowthrough
DBC: Dynamic binding capacity
HCP: Host cell protein
CV: Column volume
